# Operation for the Removal of Vicious Consolidation of Fractured Bone

**Published:** 1847-01

**Authors:** 


*Operation for the Removal of Vicious Consolidation of Fractured Bone.* By Dr. Malgaigne.—The operation had been performed on a child, aged eight years, of a strong constitution, who broke his leg at the age of twelve months ; and the fracture having been neglected, the bones united in the most irregular manner. The fracture had taken place at the union of the inferior third with the two superior thirds of the leg, and had been permitted to unite at right angles. The muscles of the posterior region of the leg were contracted, and the deformed limb was ten centimètres (3½ inches) shorter than the other. The child had been brought to M. Guersant, who refused to perform any operation; the case had been laid before the Society of Surgery in the month of July, and opinions had been very much divided on the propriety of attempting any surgical measures for the purpose of straightening the leg. On the solicitation of the family, M. Malgaigne, however, was induced to try the following plan:—The fibula being first divided with M. Liston's ostoe tome, a portion of bone in the shape of a wedge was removed from the tibia with a saw, and the periosteum of the posterior part of the bone was not divided. The bones were then straightened, and the limb placed in a fracture dressing. On the third day hospital gangrene appeared, and the child died ten days after operation.—*Ibid.*

